# Hospital referral patterns amongst older adults in Zimbabwe: a cross-sectional study

**DOI:** 10.1080/16549716.2025.2547495

**Published:** 2025-09-02

**Authors:** Jack L. Stanley, David Hettle, Rudo MS Chingono, Fadzaishe Mhino, Tsitsi Bandason, Chipo E. Mpandaguta, Karlos Madziva, Rashida A. Ferrand, Joseph Chipanga, Michael Vere, Prosper Chonzi, Justin Dixon, Celia L. Gregson, Katharina Kranzer, Ioana D. Olaru

**Affiliations:** a The Health Research Unit Zimbabwe, Biomedical Research and Training Institute, Harare, Zimbabwe; b Elizabeth Blackwell Institute, Bristol Medical School, University of Bristol, Bristol, UK; c Department of Infection, North Bristol NHS Trust, Bristol, UK; d Clinical Research Department, London School of Hygiene and Tropical Medicine, London, UK; e Department of Health, Harare City Council, Harare, Zimbabwe; f Department of Global Health and Development, London School of Hygiene and Tropical Medicine, London, UK; g Global Health and Ageing Research Unit, Bristol Medical School, University of Bristol, Bristol, UK; h Division of Infectious and Tropical Medicine, Medical Centre of the University of Munich, Munich, Germany

**Keywords:** ageing, Zimbabwe, healthcare utilisation, hospital referral, older adults

## Abstract

Over the coming decades Africa is projected to undergo a significant demographic shift towards an older population. Healthcare provision for older adults is made more complex by age-related multimorbidity and frailty, which contribute to older adults more frequently requiring intensive, hospital-based treatment than those in younger age groups. We investigate age and sex-stratified, diagnosis-specific hospital referral patterns in Harare, Zimbabwe to understand referral practices for older adults. This retrospective analysis of attendance records from primary health clinics (*n* = 8) over six years (2016 to 2021) investigated associations between age, sex and diagnosis and recommended hospital referral. Analysis compared the percentage referred between those aged ≥65 years and those younger than 65 years. The records contained 195,999 attendances. Median attendee age was 9 years (IQR 1.75–32); 52.5% were female; 5.4% were aged ≥65 years. Overall, 14.9% attendances resulted in hospital referral. The highest referral percentage by diagnosis was for trauma (47.8% referred overall, 40.5% of those aged ≥65 years referred). The overall percentage referred in those aged ≥65 years (18.5%) was the same as those aged 35–44 years (18.0%); this pattern was observed across diverse diagnoses including acute respiratory infections, hypertension and musculo-skeletal pain. Despite age-associated morbidity and theoretically free public healthcare to those aged ≥65 years in Zimbabwe, older adults are no more likely to be referred than young adults to higher level care, across multiple disease classes, including infective, musculoskeletal and cardiovascular diseases. This may reflect a healthcare system not yet orientated towards an ageing population’s needs.

## Paper context

- **Main findings**: Older adults are not prioritised for hospital referral in Harare, Zimbabwe as compared to their younger counterparts.
- **Added knowledge**: This is the first study of its kind on healthcare access amongst older adults in this area.
- **Global health impact for policy and action**: This study draws attention to the possibility that older adults face barriers to accessing hospital care, raising the importance of redoubling efforts to orient healthcare systems in the region to the challenge of ageing populations.

## Background

Rising life expectancy is causing a demographic transition across Africa, with the older population (≥60 years) projected to rise from 54 million to 161 million between 2020 and 2050 [1]. Age-associated diseases, multimorbidity and frailty change healthcare demands within a population [2], as seen with the rise in non-communicable diseases (NCDs) across Africa, over the last 30 years [3,4]. In the region, older adults commonly remain economically active [5], with half of those aged ≥65 years of age remaining in the labour force, the highest figure worldwide [6].

Healthcare delivery now needs to transition from predominantly infectious disease care to the detection and management of chronic NCDs [5] and care for an increasingly multimorbid, ageing population [7,8]. While many countries in Africa, including Zimbabwe have developed ageing-specific national health policies, few have mobilised the resources or workforce to effectively implement these [9].

Older adults can face accessibility issues related to age-related physical and communication difficulties [10], a lack of personnel specially trained in the care of older people [11], and financial barriers when accessing healthcare related to out-of-pocket costs and inadequate insurance coverage [9,12]. Furthermore ageism, a recognised phenomenon worldwide, can present additional barriers once older adults manage to access healthcare, including negative attitudes, ignorance of older patients’ opinions and lack of appropriate specialist services [11,13,14].

In Zimbabwe, older adults are entitled to free medical care at government hospitals and primary health clinics (PHCs) [15] yet underfunding and scarce physical and human resources place these services under pressure and limit accessibility [16].

Healthcare utilisation, costs and hospital referrals are highest in later life in high and upper-middle income settings [1,17]. Given the burden of NCDs and poverty in Africa, it may be expected that this effect be further pronounced, and we would hypothesise that if services were perfectly accessible, adult healthcare utilisation would be highest amongst the oldest age groups. Yet, in the published studies from low- and middle-income countries (LMICs) this is not seen [1]. This study aimed to:

1. Investigate health service use in Zimbabwe, by age and sex
2. Understand primary care-based referral practices for older adults
3. Identify any disparities in care between older adults and the rest of the population.

## Methods

### Study design and setting

We performed a retrospective analysis of data collected primarily as part of a study assessing the effect of a conjugate typhoid vaccine on antibiotic prescribing [18]. The data comprised medical records of all consultations at eight primary healthcare clinics (PHC) in Harare, between 1 January 2016 and 31 December 2021. Harare is the capital city of Zimbabwe and has a population of approximately 1.49 million, of whom 700,000 are served by the PHCs covered in our data [19]. PHCs represent the first point of medical contact for people living within their catchment areas. The clinics provide free healthcare to those ≤5 and ≥65 years old, with consultation fees charged for the remainder. Hospital referral from PHCs is recommended for severe cases, or those requiring complex, secondary care treatment.

### Analysis

Data were analysed in R version 4.4.2 [20]. Emergency hospital referral, as a binary variable, was summarised by age, sex and diagnosis. Diagnoses were categorised with diagnoses accounting for <2% of individuals classified as ‘other’ and those with no documented diagnosis to ‘unknown’. Missing data were coded as ‘NA’ (not available). In this analysis, NCDs include heart failure, cardiovascular disease, central nervous system pathology, diabetes, chronic respiratory disease and malnutrition.

Zimbabwe census data (2022) were used to determine the representativeness of the study dataset by comparing against the age/sex structure of the Harare population [19].

## Results

The dataset contained 195,999 attendances where hospital referral status (yes or no) was recorded over the six-year study period. Missing data were few for sex (< 0.01% missing), age (3.49%), attendance date (0%) and hospital referral (1.94%). Diagnosis was recorded as unknown in 11.1%, which may include both missing data and instances where a diagnosis could not be reached.

Median attendee age was 9 (IQR 1.75–32) years and 52.5% were female. The most common diagnosis across the dataset was gastroenteritis (16.1% of attendances), whilst for those aged ≥65 years it was musculo-skeletal pain (19.6% of attendances). Overall, 14.9% of attendances (*n* = 29,204) resulted in a hospital referral. Overall, females were slightly more frequently referred (15.1%) than males (14.6%). The diagnosis with the highest referral percentage was trauma both in the whole dataset (47.8% referred) and those aged ≥65 years (40.5% referred).

**Figure 1.**
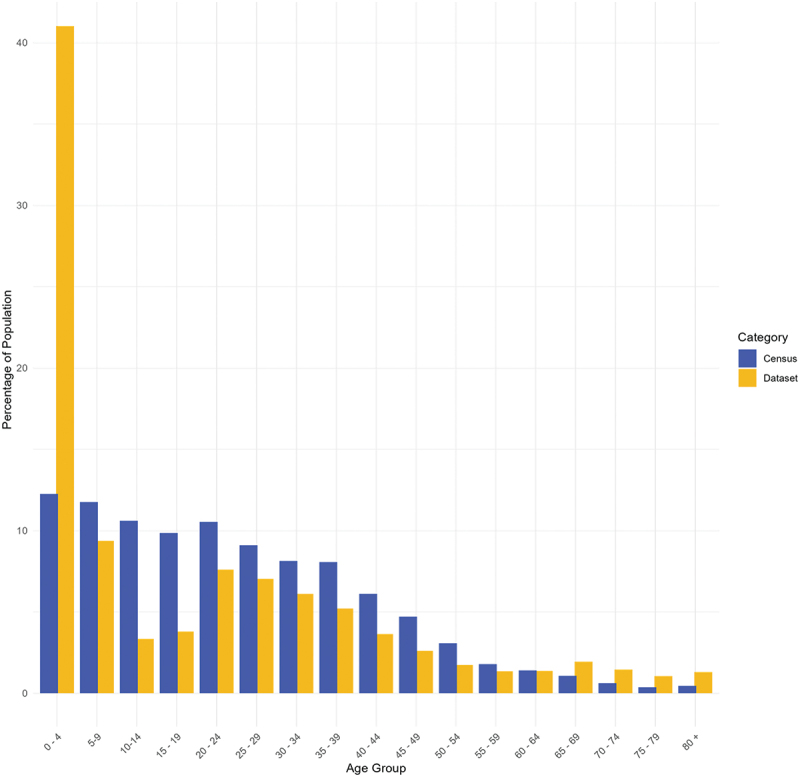
Age distribution of our population and the Zimbabwean census. Age categories mirror the 2022 Zimbabwean Census. Percentages reflect the proportion of the population in either the census (blue) or the dataset we present (yellow) within each age group. N ranges here from 79,149 (0–4 years of age) to 2525 (80+ years of age) for our dataset.

Attendees were heavily skewed towards young children, with 41.0% under five years of age. This markedly exceeded the proportion of the population in this age group (Figure 1, 12.3% of population in census <5 years of age). In total, 5.4% attendees (*n* = 10,665) were aged ≥65 years; to a lesser extent they were over-represented in comparison to their population representation (individuals aged ≥65 years make up 2.5% of the census population).

**Figure 2.**
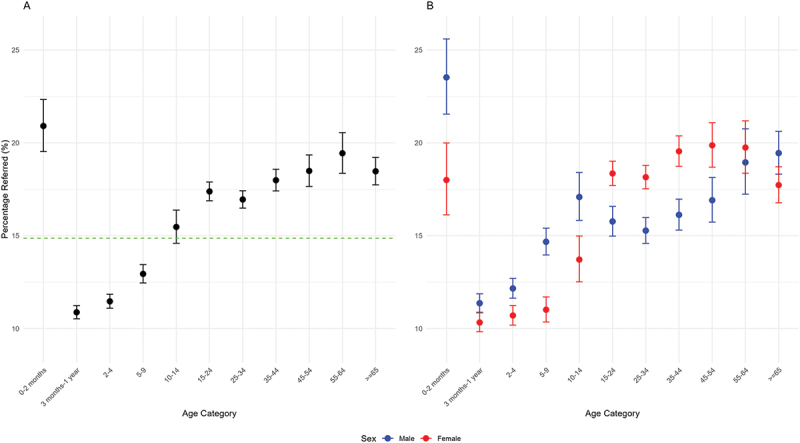
Percentage of attendances referred to hospital l by age group and sex. A – Age groups across both sexes. Green line represents mean referral across the whole population. B – Age groups divided by sex. N here (overall rather than sex stratified) is the lowest for 0–2 months (*n* = 3295). Outwith this group it ranges from 5097 for the 55–64 years of age group to 29,868 for the 3 months to 1 year group. In the ≥65 years group *n* = 10665.

Beyond the age of two months hospital referral generally increased with age (from 11.2% at three months-one year to 18.5% in those aged ≥65 years) (Figure 2A). However, the proportion of those aged ≥65 years who were referred (18.5%) was similar to those aged 35–44 years (18.0%). Stratification by sex and age group (Figure 2B) showed that up to the age of 14 years males were more frequently referred than females. From 15–54 years the reverse was true. Beyond the age of 55 years there was no difference in referral by sex alone.

**Figure 3.**
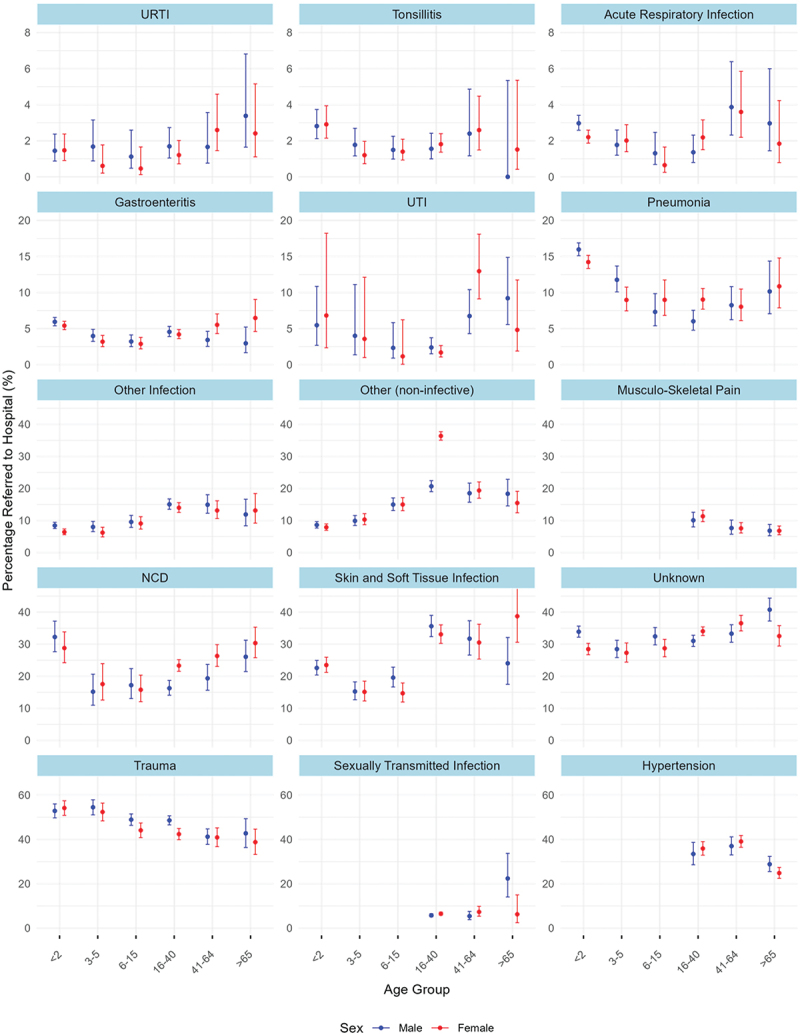
Percentage of attendances referred to hospital by age group, sex and diagnosis. Diagnosis is labelled directly above each plot. Age category (x axis) is shared across all plots. Y axes are shared row-wise. Hypertension, STI and Musculo-skeletal pain excluded for <15 years due to low case numbers in these groups and dubious clinical relevance of such diagnoses in these age groups. Number of overall cases by diagnosis (ie those referred and not referred) is as follows: URTI (7652), Tonsillitis (13742), Acute Respiratory Infection (23326), Gastroenteritis (31537), UTI (3198), Pneumonia (20786), Other Infection (15825), Other (non-infective) (21158), NCD (6585), Skin and Soft Tissue Infection (7757), Unknown (22172), Trauma (11535), Sexually Transmitted Infection (10507), Hypertension (5265).

Of those attending with acute respiratory infection (ARI), a smaller proportion of those aged ≥65 years were referred to hospital than those aged 41–64 years (Figure 3). This pattern was also seen for trauma and hypertension. For pneumonia and NCDs a slightly higher proportion of attendances in those aged ≥65 years were referred than seen in those aged 41–64 years.

When stratified by diagnosis, sex differences were noted for skin and soft tissue infection and gastroenteritis where a greater proportion of women than men aged ≥65 years were referred, whilst for urinary tract infection (UTI) the reverse was true.

## Discussion

This study demonstrates that referrals from primary to higher-level healthcare for older adults (≥65 years) in Harare, Zimbabwe, are no higher than for younger adults. This pattern is evident across multiple diagnostic categories, including infections and trauma. This is despite a disproportionately high PHC attendance for the number of older people in the population, reflecting greater health seeking from this multimorbid age group. As anticipated, infants frequently attended PHCs, but unlike older adults, were often referred to hospital. This discrepancy is contrary to our expectation that older adults would be referred to hospital more often than younger adults, owing to age-related factors complicating treatment such as polypharmacy, frailty and multimorbidity.

Except for pneumonia (and soft tissue infection in women) there was no increase in infection referrals in the oldest age group. Yet, older adults are more vulnerable to infections and their consequences, with worse outcomes compared to younger people [21], necessitating more intense management, warranting referral to higher levels of care [22]. Regarding trauma and other musculoskeletal conditions, again there was no increase in referral in the older group, despite high rates of falls, fragility fractures [23] and injuries [24], and referrals for musculoskeletal pain fell sharply with age, suggesting a ‘normalisation’ of age-associated pain.

The disproportionately low referrals among older people may be explained by high out-of-pocket costs of health care and relative de-prioritisation of the health of older people, whose economic value may be perceived as low. Furthermore, very few healthcare personnel have received specific training in relation to ageing health, and within Zimbabwe medical insurance coverage is minimal [12]. Few studies have investigated age-specific healthcare provision in Africa; none in Zimbabwe. The WHO has acknowledged that ageing-appropriate services are most lacking in LMICs [1]. Healthcare systems which have historically focused on single diseases may be less well equipped to manage multimorbidity, which is more commonly seen in older attendees [7,25].

Our extensive collection of routine clinical data spanning 6-years, is a strength. Due to its unique nature, there are few comparative data published. Study limitations include the retrospective observational design reliant upon paper-based documentation from clinics, hence data are likely incomplete, although most likely non-differential by age or sex. Data reflect referrals made, not referral attendances nor the referral nature (e.g routine outpatient appointment or emergency referrals).The analysis focuses on single disease attendances (reflecting PHC records) overlooking multimorbidity, which is not routinely reported. Our work reflects the Zimbabwean context only and future work should investigate other settings in Africa.

This study suggests that older adults are de-prioritised for hospital referral in Zimbabwe. Rising longevity and age-related NCDs warrant greater recognition of older adults’ complex healthcare needs. It is critical policymakers consider why secondary care referrals do not mirror the increasing health needs of older adults. Future work must include qualitative exploration of barriers to accessing health services and investigation of whether multimorbidity influences referral patterns, to inform guideline development, training to improve recognition of the complexity of medical care for older adults and more equitably accessible health services.

## Supplementary Material

GHA_STROBE_Checklist_Ageing_Zimbabwe.docx

## Data Availability

Individual de-identified participant data and data dictionaries will be made available on Dryad (www.datadryad.org) within 12 months of publication. Prior to these interested parties may contact the corresponding author.
