# Supplementary material for: Hospital referral patterns amongst older adults in Zimbabwe: a cross-sectional study
Source: Glob Health Action. 2025 Sep 2;18(1):2547495. doi: 10.1080/16549716.2025.2547495 (PMC12406308; doi:10.1080/16549716.2025.2547495)
Supplement: GHA_STROBE_Checklist_Ageing_Zimbabwe.docx [file ZGHA_A_2547495_SM5192.docx]

STROBE Statement—Checklist of items that should be included in reports of ***cross-sectional studies***

Study name: Hospital referral patterns amongst older adults in Zimbabwe: A cross-sectional study.

Authors: Jack L Stanley^1,2,4^, David Hettle^1,2,4^, Rudo MS Chingono^1^, Fadzaishe Mhino^1^, Tsitsi Bandason^1^, Chipo E. Mpandaguta^1^, Karlos Madziva^1^, Rashida A. Ferrand^1,3^, Joseph Chipanga^1^, Michael Vere^6^, Prosper Chonzi^6^, Justin Dixon^1,7^, Celia L Gregson^1,5^, Katharina Kranzer^1,3,8^, Ioana D Olaru^1,3^.

Affiliations

1 The Health Research Unit Zimbabwe, Biomedical Research and Training Institute, Harare, Zimbabwe

2 Elizabeth Blackwell Institute, Bristol Medical School, University of Bristol, Bristol UK

3 Clinical Research Department, London School of Hygiene and Tropical Medicine, London, United Kingdom

4 North Bristol NHS Trust, Bristol, UK

5 Global Health and Ageing Research Unit, Bristol Medical School, University of Bristol, UK

6 Department of Health, Harare City Council, Harare, Zimbabwe

7 Department of Global Health and Development, London School of Hygiene and Tropical Medicine, London, United Kingdom

8 Division of Infectious and Tropical Medicine, Medical Centre of the University of Munich, Munich, Germany

Date: 20/6/2025

|  | Item No | Recommendation | Present in manuscript | |
| --- | --- | --- | --- | --- |
| **Title and abstract** | 1 | (*a*) Indicate the study’s design with a commonly used term in the title or the abstract | 🗸 Line 1 |  |
|  |  | (*b*) Provide in the abstract an informative and balanced summary of what was done and what was found | 🗸 Line 34-56 |  |
| Introduction | | |  |  |
| Background/rationale | 2 | Explain the scientific background and rationale for the investigation being reported | 🗸 Line 58-64 |  |
| Objectives | 3 | State specific objectives, including any prespecified hypotheses | 🗸 Lines 87-93 |  |
| Methods | | |  |  |
| Study design | 4 | Present key elements of study design early in the paper | 🗸 Lines 106-108 |  |
| Setting | 5 | Describe the setting, locations, and relevant dates, including periods of recruitment, exposure, follow-up, and data collection | 🗸 Line 96-100 |  |
| Participants | 6 | (*a*) Give the eligibility criteria, and the sources and methods of selection of participants | 🗸 Line 98 |  |
| Variables | 7 | Clearly define all outcomes, exposures, predictors, potential confounders, and effect modifiers. Give diagnostic criteria, if applicable | 🗸Lines 106-111 |  |
| Data sources/ measurement | 8* | For each variable of interest, give sources of data and details of methods of assessment (measurement). Describe comparability of assessment methods if there is more than one group | 🗸 Line 98 |  |
| Bias | 9 | Describe any efforts to address potential sources of bias | 🗸Lines 112-113 |  |
| Study size | 10 | Explain how the study size was arrived at | 🗸 Line 98 |  |
| Quantitative variables | 11 | Explain how quantitative variables were handled in the analyses. If applicable, describe which groupings were chosen and why | 🗸 Lines 106-111 |  |
| Statistical methods | 12 | (*a*) Describe all statistical methods, including those used to control for confounding | 🗸 Lines 106-111 |  |
|  |  | (*b*) Describe any methods used to examine subgroups and interactions | 🗸 Lines 106-111 |  |
|  |  | (*c*) Explain how missing data were addressed | 🗸 Line 109 |  |
|  |  | (*d*) If applicable, describe analytical methods taking account of sampling strategy | n/a |  |
|  |  | (*e*) Describe any sensitivity analyses | n/a |  |
| Results | | |  |  |
| Participants | 13* | (a) Report numbers of individuals at each stage of study—eg numbers potentially eligible, examined for eligibility, confirmed eligible, included in the study, completing follow-up, and analysed | n/a |  |
|  |  | (b) Give reasons for non-participation at each stage | n/a |  |
|  |  | (c) Consider use of a flow diagram | n/a |  |
| Descriptive data | 14* | (a) Give characteristics of study participants (eg demographic, clinical, social) and information on exposures and potential confounders | 🗸 Lines 120-126 |  |
|  |  | (b) Indicate number of participants with missing data for each variable of interest | 🗸 Lines 116-118 |  |
| Outcome data | 15* | Report numbers of outcome events or summary measures | 🗸 Line 123 |  |
| Main results | 16 | (*a*) Give unadjusted estimates and, if applicable, confounder-adjusted estimates and their precision (eg, 95% confidence interval). Make clear which confounders were adjusted for and why they were included | n/a |  |
|  |  | (*b*) Report category boundaries when continuous variables were categorized | 🗸 |  |
|  |  | (*c*) If relevant, consider translating estimates of relative risk into absolute risk for a meaningful time period | n/a |  |
| Other analyses | 17 | Report other analyses done—eg analyses of subgroups and interactions, and sensitivity analyses | n/a |  |
| Discussion | | |  |  |
| Key results | 18 | Summarise key results with reference to study objectives | 🗸 Lines 156-162 |  |
| Limitations | 19 | Discuss limitations of the study, taking into account sources of potential bias or imprecision. Discuss both direction and magnitude of any potential bias | 🗸 Lines 183-185 |  |
| Interpretation | 20 | Give a cautious overall interpretation of results considering objectives, limitations, multiplicity of analyses, results from similar studies, and other relevant evidence | 🗸 Lines 189-195 |  |
| Generalisability | 21 | Discuss the generalisability (external validity) of the study results | 🗸 Lines 188-189 |  |
| Other information | | |  |  |
| Funding | 22 | Give the source of funding and the role of the funders for the present study and, if applicable, for the original study on which the present article is based | 🗸 Lines 198-208 |  |

*Give information separately for exposed and unexposed groups.

**Note:** An Explanation and Elaboration article discusses each checklist item and gives methodological background and published examples of transparent reporting. The STROBE checklist is best used in conjunction with this article (freely available on the Web sites of PLoS Medicine at http://www.plosmedicine.org/, Annals of Internal Medicine at http://www.annals.org/, and Epidemiology at http://www.epidem.com/). Information on the STROBE Initiative is available at www.strobe-statement.org.
